# Innovative Connection of Non-Load-Bearing Walls Using a Spatially Arranged Silica Glass Mesh

**DOI:** 10.3390/ma19132900

**Published:** 2026-07-06

**Authors:** Radosław Jasiński, Iwona Galman

**Affiliations:** 1 Department of Building Structures, Silesian University of Technology, Akademicka 5, 44-100 Gliwice, Poland; radoslaw.jasinski@polsl.pl; 2 Department of Structural Engineering, Silesian University of Technology, Akademicka 5, 44-100 Gliwice, Poland

**Keywords:** masonry structures, AAC masonry units, wall joints, connectors, Silica Glass Mesh

## Abstract

**Highlights:**

- An innovative connection for non-load-bearing walls using spatially arranged Sil-ica Glass Mesh (SGM) is proposed.
- Original test results of wall connections subjected to monotonically increasing in-plane shear loading using a spatially arranged mesh are presented, and the re-sults are compared with both the traditional bonding solution and a flat mesh placed in the bed joints.
- It is demonstrated that the novel mesh configuration positively influences the connection parameters, enabling effective utilisation of the mesh tensile capacity.
- A preliminary standard mechanical model of the connection is developed, allow-ing the prediction of forces and displacements with an acceptable safety margin; however, further research is required.
- The proposed method appears promising for practical application, is economically justified, and provides an effective solution for connecting non-load-bearing walls, serving as an alternative to traditional bonding.

**Abstract:**

Although non-structural walls do not determine the structural safety of a building, they are responsible for its functionality by serving as acoustic, thermal, and fire-resistant partitions. They may be freely located and relocated and are typically constructed during the finishing stage of building works. Reliable performance of non-structural walls depends on appropriate connections to floors and adjacent walls. Connections to walls are most commonly achieved using traditional masonry bonding or sufficiently durable wall connectors, usually made of steel. An alternative to steel connectors may be connectors made of polymer-based materials or meshes. This paper proposes an innovative method for connecting non-structural masonry walls using a spatially arranged mesh, which serves not only as reinforcement of the wall connection but also as reinforcement of the bed joints. The aim of the study was to evaluate the effectiveness of this method in comparison with other connection techniques, including traditional solutions. Experimental investigations were carried out using an original test setup on 12 specimens made of AAC masonry units, divided into three series: series P—traditional connection (reference series), series H—connection with mesh placed in bed joints, and series SHP—connection with spatially arranged mesh. Silica Glass Mesh (SGM), intended for reinforcement of bed joints in AAC masonry, was used in the study. The experiments focused on the analysis of connection behavior and load-bearing capacity, with particular emphasis on maximum load values and failure mechanisms. Individual stages of the behavior of mesh-reinforced connections were identified, and empirical relationships enabling estimation of maximum loads were developed. The results confirmed that the traditional connection achieved the highest load-bearing capacity. However, as expected, the mesh-reinforced connections—particularly those with the spatial mesh arrangement—exhibited a more stable response and a greater ability for progressive load transfer. The SHP series connections with spatially arranged meshes exhibited significantly lower load-bearing capacity compared to the reference unreinforced connections, while at the same time demonstrating substantially greater deformability. The stiffness degradation in the mesh-reinforced connections did not occur abruptly, as observed in the reference models, which makes them an effective alternative for practical applications. Technical models for predicting forces and displacements of connections reinforced with spatially arranged meshes and meshes placed in bed joints were also developed.

## 1. Introduction

The literature on masonry structures emphasizes that the safety of a building depends not only on the load-bearing capacity of individual walls, but also on the quality of their mutual connections. The lack of proper wall bonding leads to the loss of three-dimensional structural action (so-called box action), resulting in increased susceptibility to horizontal actions, particularly seismic and wind loads. Enhancement of connection stiffness and wall-to-wall load-bearing capacity is crucial for the global stiffness and load-bearing performance of masonry buildings [1,2].

The issue of masonry wall connections remains insufficiently investigated and underestimated, both with respect to load-bearing and non-load-bearing walls.

In addition to the use of confined masonry technology, in which reinforced concrete tie-columns are applied at wall intersections to provide rigid wall-to-wall connections [3,4,5,6], steel connectors recommended by standards are also commonly used [7]. The effectiveness of steel connectors has been confirmed, among others, by the studies presented in [8,9,10,11,12].

Connectors in the form of perforated steel strips or steel angles provide approximately 25% of the maximum load capacity achieved by traditional masonry bonding, while simultaneously increasing the ductility of the connections, which is undoubtedly advantageous under cyclic and seismic loading conditions. However, a significant disadvantage of steel connectors is the necessity for careful installation and provision of sufficient anchorage length within bed joints or masonry units. Furthermore, the connectors must be manufactured from corrosion-resistant steel or protected against corrosion [7], which substantially increases application costs.

Steel connectors [13] do not always meet expectations in terms of load-bearing capacity, serviceability, and durability, particularly in view of the limited availability of comprehensive experimental data and the lack of standardized testing procedures

An alternative to steel wall connectors is the use of non-metallic connectors. In recent years, strengthening methods employing non-metallic materials have been intensively developed, particularly systems based on glass fiber-reinforced polymers (GFRP), carbon fiber-reinforced polymers (CFRPs), and basalt fiber-reinforced polymers (BFRP). Experimental studies have demonstrated that the application of mortars reinforced with GFRP meshes leads to a significant increase in the load-bearing capacity and deformability of masonry walls, especially under in-plane shear loading conditions [14,15].

In the study by Gattesco and Boem [14], it was shown that the presence of the mesh limits crack propagation and modifies the failure mechanism, while the investigations presented in [15] confirmed improvements in strength parameters in diagonal compression tests. A further development of this technology includes TRM (Textile-Reinforced Mortar) and FRCM (Fabric-Reinforced Cementitious Matrix) systems, in which fiber meshes are embedded within a mineral mortar matrix. Research indicates that these systems improve not only the strength but also the ductility of masonry structures, which significantly enhances structural safety and reliability [16]. In particular, a transition from brittle to more gradual failure mechanisms has been observed.

In the context of wall-to-wall connections, ensuring continuity of reinforcement within corner zones and wall intersections is of key importance. Research results indicate that surface strengthening of a single wall alone does not guarantee improved three-dimensional structural behavior unless the reinforcement is properly anchored or extended through the wall intersection zone [17]. Particular emphasis is placed on construction details such as anchorage length and the method of guiding meshes through corners.

In engineering practice, these solutions have evolved into CRM (Composite-Reinforced Mortar) systems, in which, in addition to meshes, non-metallic connectors passing through the wall thickness are also used. These connectors ensure composite action between the strengthening layer and the substrate and enable effective integration of walls within their connection zones [18]. This approach treats the strengthening solution as a complete system composed of mesh–mortar–connector interaction.

In summary, the use of meshes and non-metallic materials constitutes a promising method for reinforcing connections between masonry walls, including non-structural walls. However, their effectiveness depends on ensuring continuity of reinforcement and adequate anchorage within wall intersection zones. At the same time, there is a clear research gap regarding the understanding and description of the behavior of such structural elements. The current state of knowledge does not even allow for an approximate assessment of safety due to the lack of any experimental references.

This paper presents an innovative method for connecting non-structural masonry walls using spatially arranged Silica Glass Mesh (SGM). The proposed solution is a natural consequence of the simultaneous use of bed-joint reinforcement and wall connection reinforcement. The concept is based on appropriate shaping of the ends of polymer mesh strips (serving as bed-joint reinforcement) in such a way that they provide a connection between adjacent walls, while the mesh, in accordance with its intended function, works exclusively in tension. The novelty of the proposed solution lies in the utilization of the mesh’s mechanical properties through proper anchorage of the mesh ends within the bed joints. As a result, the need for additional wall connectors in walls reinforced within bed joints has been eliminated. The applied solution, by combining traditional bed-joint reinforcement with wall connectors, makes it possible to reduce the reinforcement costs of non-structural walls by as much as 50–100%.

The primary objective of the conducted research was to evaluate the effectiveness of the proposed method in comparison with other connection techniques, including traditional solutions. Original experimental investigations were carried out using a proprietary test setup [12,19]. The experimental program included three series of specimens (12 test elements in total): series P—traditional connection adopted as the reference solution, series H—connection reinforced with Silica Glass Mesh (SGM) placed within bed joints, and series SHP—connection reinforced with spatially arranged SGM. The experiments focused on the analysis of connection behavior and load-bearing capacity, with particular emphasis on maximum load values and failure mechanisms.

A significant novelty of the presented study is the experimental investigation conducted using an original testing setup for wall connections reinforced with appropriately shaped bed-joint reinforcement instead of traditional masonry bonding or steel connectors. The study fills a clear research gap in the field of investigations and analyses of wall connections reinforced with alternative reinforcement systems compared to conventional steel reinforcement. A qualitative description of the behavior of connections reinforced with mesh placed within bed joints and with spatially arranged mesh was presented. Similarities and differences in the behavior of mesh-reinforced connections relative to traditional masonry connections and connections reinforced with mesh embedded in bed joints were identified and discussed.

## 2. Code Requirements for the Connection of Non-Structural Walls

According to the provisions of Eurocode 6 [20], masonry walls arranged at right or oblique angles to each other shall be connected in a manner ensuring effective transfer of both vertical loads (e.g., self-weight and imposed loads) and horizontal forces resulting from wind action, earth pressure, or seismic effects. This requirement arises from the necessity to ensure the continuity and structural integrity of the building, as well as to maintain adequate global stiffness.

In engineering practice, a range of technical solutions is employed to satisfy this requirement. The most commonly used methods include traditional masonry bonding, in which elements of adjacent walls are interlocked during construction, as well as the use of steel connectors (typically in the form of ties or bars), or spatial reinforcement embedded and properly anchored in both connected walls. These solutions enable the transfer of forces between structural elements and limit the occurrence of relative displacements at the wall interface.

**Figure 1:**
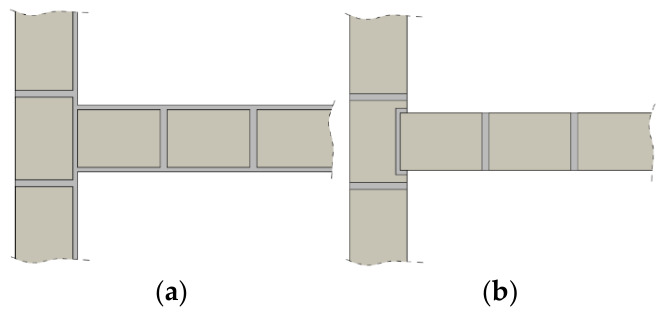
Joints in non-load-bearing walls: (**a**) with the use of plaster, (**b**) dovetail joint.

In the case of non-load-bearing walls, which primarily serve as architectural partitions and do not participate in the transfer of significant structural loads (apart from self-weight), simplified solutions are permitted. Due to the fact that their potential removal does not significantly affect the ultimate limit state of the overall structural system, alternative connection methods may be applied. In addition to the aforementioned conventional techniques, practical applications also include plaster-based connections (Figure 1a), in which the infill material acts as a bonding element, as well as mechanical interlocking solutions such as the so-called “dovetail” joint (Figure 1b), involving the formation of an appropriately shaped groove in the adjacent wall to enable anchorage of the perpendicular wall.

However, it should be clearly emphasized that butt joints executed without interlocking of masonry units or without mechanical connectors do not provide any effective support in the vertical plane. Their function is limited solely to restraining horizontal displacements, which significantly restricts their applicability in the context of structural load transfer. From the perspective of fire safety, standard [20] explicitly recommends the use of metal connectors for vertical joints between non-load-bearing and load-bearing walls. The application of such solutions enhances the stability of the partition under fire conditions, prevents premature failure, and thereby contributes to achieving the primary objective of ensuring occupant safety in emergency situations.

## 3. Proposed New Connection Method

In currently available solutions for wall connections, there is a clear lack of methods dedicated to non-structural walls that would simultaneously ensure effective in-plane support while meeting practical requirements such as ease of installation and relatively low cost. Existing approaches often represent a compromise between load-bearing capacity and simplicity of execution, which in many cases limits their applicability in engineering practice, particularly with regard to non-load-bearing partitions.

Studies on wall connections using the most common connectors available on the market, reported in [19,21], have not confirmed the assumption that such connectors can effectively replace traditional masonry bonding. In particular, it has been demonstrated that, in the case of connections made using steel connectors, the achieved load-bearing capacity was significantly lower, reaching reductions of up to approximately 35% compared to classical masonry bonding. Moreover, unfavorable phenomena associated with connector deformation were observed, whereby the connectors, under load, penetrated into the masonry unit, causing local crushing and secondary damage to the material. These effects negatively influence not only the load-bearing capacity of the connection but also its durability and long-term reliability.

In response to these limitations, an attempt was made to modify existing solutions by altering the configuration of the reinforcement mesh. The objective was to develop a connection method that would, on the one hand, limit horizontal displacements of the walls and, on the other hand, provide effective vertical support, which is of key importance for the behavior of non-load-bearing walls. An additional requirement was to maintain structural simplicity and ease of installation, which are essential for practical implementation under construction conditions.

Moreover, the proposed concept assumes the utilisation of tensile action in the mesh elements, enabling a more effective transfer of tensile forces within the connection. This approach allows for an increase in load-bearing capacity compared to meshes placed exclusively in the bed joints, while simultaneously reducing stress concentrations and improving the overall mechanical response of the connection. As a result, the proposed solution represents an attempt to combine the advantages of traditional masonry techniques with a modern material and structural approach (based on non-metallic meshes), addressing the identified shortcomings of existing technologies used in non-load-bearing walls.

The proposed connection is made of a reinforcement mesh intended for bed joint reinforcement in masonry, in which, at relative deformations exceeding 1%, a conventional or distinct yield limit of the mesh fibers is achieved. The mesh used in the bed joints and within the connection is arranged spatially.

The mesh sheet for bed joint reinforcement (1) in the connected wall (6) is placed over the entire surface of the bed joint. Subsequently, in the plane of the wall connection, the mesh is cut along its longitudinal axis. The upper horizontal anchorage strip and the upper vertical strip (2, 4) are bent into the bed joint of the layer above, while the lower horizontal anchorage strip and the lower vertical strip (3, 5) are bent into the layer below of the adjoining wall (7).

The vertical parts of the connector (4, 5), passing through the vertical joint of the connection, should be pre-tensioned (without any slack) and are recommended to be inclined at an angle of 95° relative to the plane of the bed joint. The length of the horizontal parts of the connector (1, 2, 3) should correspond to the minimum anchorage length, but not less than 38 cm. Otherwise, mechanical anchorage of the mesh to the masonry unit is required.

The horizontal parts of the connector should be embedded in mortar with an upper and lower layer thickness of not less than 1 mm (thin-layer mortar cover) and 5 mm (general-purpose mortar cover—standard thickness joints), with a tolerance of ±0.5 mm.

**Figure 2:**
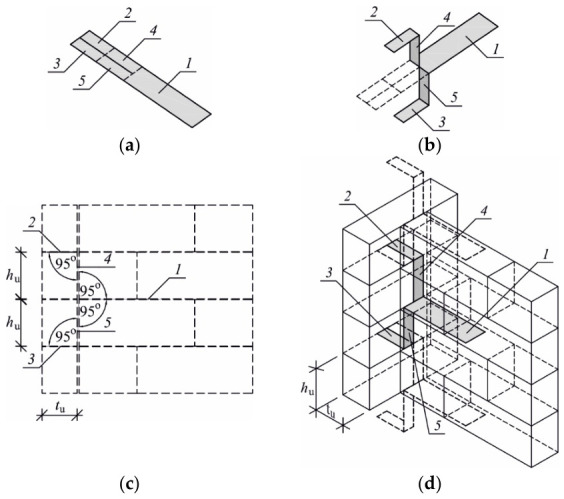
Concept of spatial mesh arrangement: (**a**) shape of the reinforcement mesh prepared for installation, (**b**) view of the spatial arrangement of the mesh within the mortar joints, (**c**) schematic layout of the mesh in the two connected walls, (**d**) spatial view of the mesh arrangement in the connected walls. (description in the text).

This method of wall connection is suitable for bonding walls made of masonry units of groups 1, 2, and 4, using both normal-thickness and thin-layer joints. The mesh used for connecting non-structural walls is spatially arranged within three bed joints and the vertical joint of the connection. The arrangement of the proposed innovative connection is illustrated in Figure 2.

## 4. Original Experimental Research

### 4.1. Experimental Program

Three series of specimens (a total of 12 test models) with identical geometry and dimensions were constructed and tested. The models were monosymmetric, T-shaped elements, in which both the web and the flange had lengths of approximately 89 cm. A vertical connection was formed between the loaded and unloaded walls, the configuration of which was intentionally varied.

**Figure 3:**
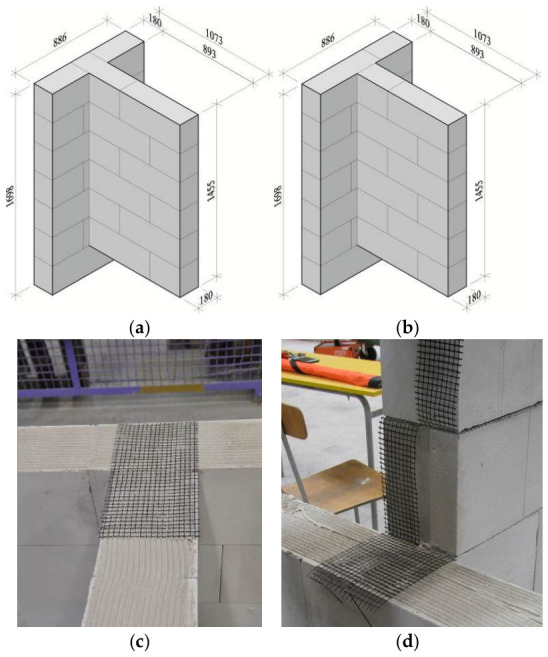
Geometry and details of the test models: (**a**) geometry of the series P models with traditional masonry bonding adapted from Ref. [19]; (**b**) geometry of the mesh-reinforced models of series H and SHP adapted from Ref. [19]; (**c**) details of flat mesh connection method—series H; (**d**) connection method using the innovative spatial arrangement of meshes—series SHP [dimensions in mm].

In the series of test models denoted as P, a traditional masonry connection was executed between the flange and the web (Figure 3a). These elements were treated as reference models, whose mechanical parameters and behavior under loading and failure were compared with the results obtained from the remaining test series. A total of six reference models were prepared.

In the other two series (three test models per series), the connection between the flange and the web of the test model was achieved using a mesh (wall geometry according to Figure 3b). In series H, a single flat-laid mesh was applied (Figure 3c), whereas in series SHP, the mesh was arranged in an innovative spatial configuration (Figure 3d).

### 4.2. Materials

The specimens were constructed using autoclaved aerated concrete (AAC) masonry units bonded with system mortar of class M5, applied in thin joints, while vertical head joints were left unfilled. The compressive strength of the masonry, determined in accordance with PN-EN 1052-1:2000 [22], was *f_c_* = 2.97 N/mm^2^, and the modulus of elasticity was *E_m_* = 2040 N/mm^2^. The initial shear strength was established following PN-EN 1052-3:2004 [23] was *f_vo_* = 0.31 N/mm^2^, and the average friction coefficient in dry joints was equal to μ = 0.92. The shear modulus and masonry stiffness determined in accordance with ASTM E519-81 [24] were *G* = 329 N/mm^2^ and *K_RL_* = 117.1 MN/m, respectively.

**Figure 4:**
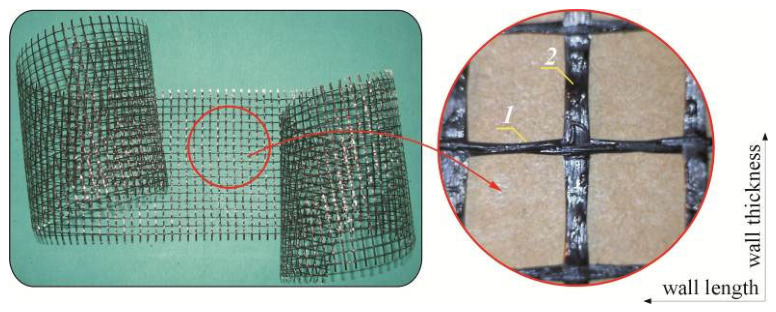
MAP-Reinforcement (SGM) bed-joint reinforcement used in the investigations: 1—warp (D1) fibres, 2—weft (D2) fibres.

Structural bed-joint reinforcement of the MAP-Reinforcement type [25], consisting of connected strands of Silica Glass Mesh (SGM) coated with a polymeric compound and having a mesh size of 10 × 10 mm (Figure 4), was used as reinforcement for the wall connections. The mesh consisted of two-twisted weft fibers with a diameter of 0.5 mm and flat warp fibers with a rectangular cross-section of 0.25 × 1.5 mm. Within the wall cross-section, the cross-sectional area of the mesh reinforcement in a single bed joint was *A_s_* = 7.07 mm^2^. Based on ASTM D-2023-2011 [26], the average tensile strengths were determined as *F_D_*_1_ = 25.1 kN/m (Warp D1) and *F_D_*_2_ = 37.6 kN/m (Weft D2), while the elongation at maximum load reached *ε_D_*_1_ = 1.99% (Warp D1) and *ε_D_*_2_ = 2.56% (Weft D2).

### 4.3. Testing Methodology

**Figure 5:**
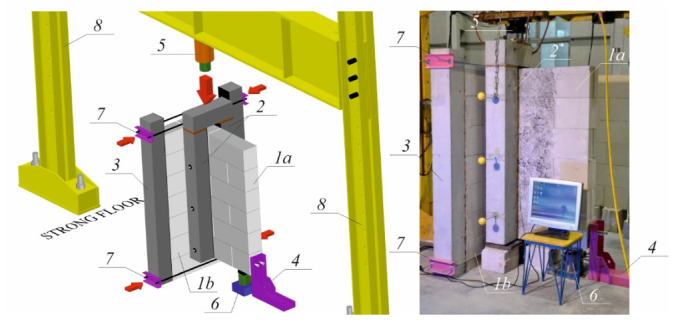
A scheme and photo of the test stand: 1a—longitudinal wall, 1b—transverse wall, 2—reinforced concrete column transferring shear load, 3—reinforced concrete pillars limiting horizontal deformation, 4—horizontal support, 5—system of the hydraulic cylinder and the force gauge used to induce shear stress, 6—force gauge, vertical reaction, 7—horizontal tie, 8—steel frame. Adapted from Ref. [19].

The experimental program was carried out using a test setup specifically designed for this purpose (Figure 5). Specimens labeled 1a and 1b, equipped with confining elements (3) and load-transferring components (2), were positioned on a rigid, strong floor (panel 1b). The system was supported by a dynamometer (6) combined with a resistor (4), forming a pinned and fixed support condition.

The specimens were placed beneath a steel frame (8), to which a hydraulic actuator with a capacity of 1000 kN was attached. This actuator applied shear loading at a constant displacement rate of 1 mm/min. The structural response was measured using an inductive force transducer with a maximum capacity of 250 kN (class 1). The uncertainty of a single force measurement was equal to *u_R_* = 0.01/$\sqrt{3}\;$*N_i_*.

To simulate the behavior of a long wall segment in panel 1b, a prestressing level of 0.1 MPa was introduced. This was achieved using reinforced concrete elements (3) together with steel tendons (7). Each specimen was subjected to monotonic loading until failure. The vertical load responsible for generating shear forces was distributed evenly along the entire height of the wall through elements (2). As a result, shear stresses in the joints were assumed to be uniformly distributed. During the tests, both load and relative displacement between the loaded and unloaded walls were continuously recorded.

Two independent data acquisition systems were employed. One side of the specimen was monitored using the ARAMIS optical system for displacement measurement, while the opposite side was instrumented with inductive displacement transducers (PJX-10), featuring a measurement range of 10 mm and an accuracy of ±0.002 mm.

### 4.4. Test Results

#### 4.4.1. Failure Mechanism

**Figure 6:**
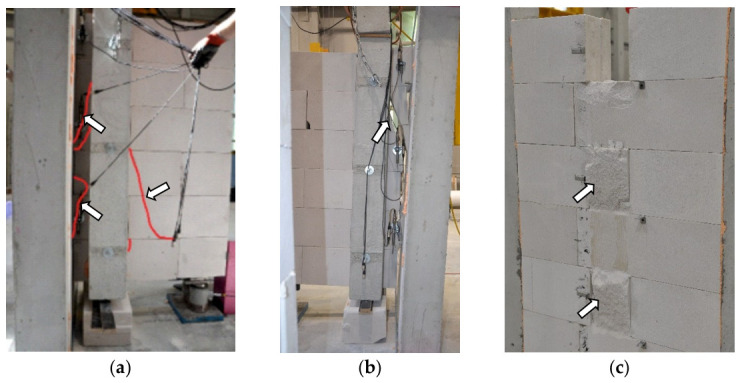
Destruction of models of series P: (**a**) a first crack on the reference model P_2, (**b**) joint after failure P_5, (**c**) joint after failure P_3.

All unreinforced specimens showed similar behavior. In the initial stage of loading, no cracking sounds or visible damage on the lateral surfaces of the elements were observed. However, localized non-dilatational deformations appeared in some areas of the wall. This stage continued until the first diagonal cracks formed near the wall joints (Figure 6a). As the load increased, the existing cracks became more pronounced, especially along the joints, and gradually propagated toward the reinforced concrete column responsible for load transfer (Figure 6b). The maximum load was reached at this stage. Further loading resulted in significant relative displacements and rotation between the connected wall segments. After failure, the joint was separated (Figure 6c). The failure was characterized by nearly vertical shearing of the elements forming the connection, while no significant damage was observed in the remaining elements.

**Figure 7:**
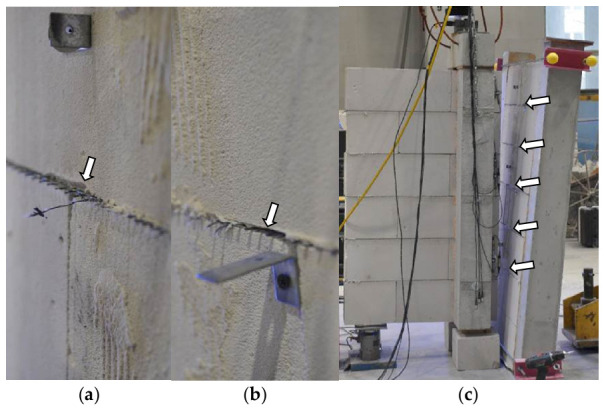
Failure of reinforced models: (**a**) mesh rupture H_1, (**b**) mesh rupture SHP_1, (**c**) separation of the two connected walls SHP_2.

In the case of type H models (connected using a single layer of mesh), the failure process was distinctly different and exhibited a dynamic character. No warning signs were observed, such as the gradual formation of cracks or audible indications of cracking. Failure occurred suddenly, without any prior symptoms indicating imminent collapse. At the moment of failure, a rapid displacement of the loaded wall relative to the unloaded wall was observed. Rupture of the mesh fibers occurred; however, the mesh was not pulled out of the mortar but instead fractured (Figure 7a).

This indicates good interaction between the mesh and the mortar, as well as effective force transfer within the connection up to the point of failure. The mesh openings filled with mortar acted as mechanical anchors, increasing adhesion and the load transfer capacity between the connected elements. At the same time, this led to an increase in system stiffness, but at the expense of limited deformation capacity, resulting in sudden, brittle failure.

For type SHP models, the failure process exhibited a more gradual, staged character. With increasing load, a progressive displacement of the loaded wall relative to the unloaded wall was observed. The course of this process was largely proportional to the increase in applied load, indicating a more predictable and less abrupt structural response.

Upon reaching the maximum load, failure of the connection occurred due to rupture of the mesh fibers (Figure 7b). Similarly to the H-type models, no pull-out of the mesh from the mortar was observed; instead, the mesh ruptured. This indicates that the failure mechanism was governed by the tensile strength of the mesh material rather than by loss of adhesion at the mesh–mortar interface.

The observed and repeatable failure mechanism clearly indicates that, in the case of the spatial mesh, the load-transfer mechanism differs from that of the horizontally placed mesh. The complete rupture of all mesh strips confirms the activation of tie-action behaviour, even though direct stress measurements within the mortar joint are not technically feasible.

In both H-type and SHP-type models, no cracking of the masonry itself was observed. The failure process did not involve damage to the masonry, but rather the separation of the two connected walls (Figure 7c).

#### 4.4.2. Load–Displacement Relationships. Basic Experimental Results

**Figure 8:**
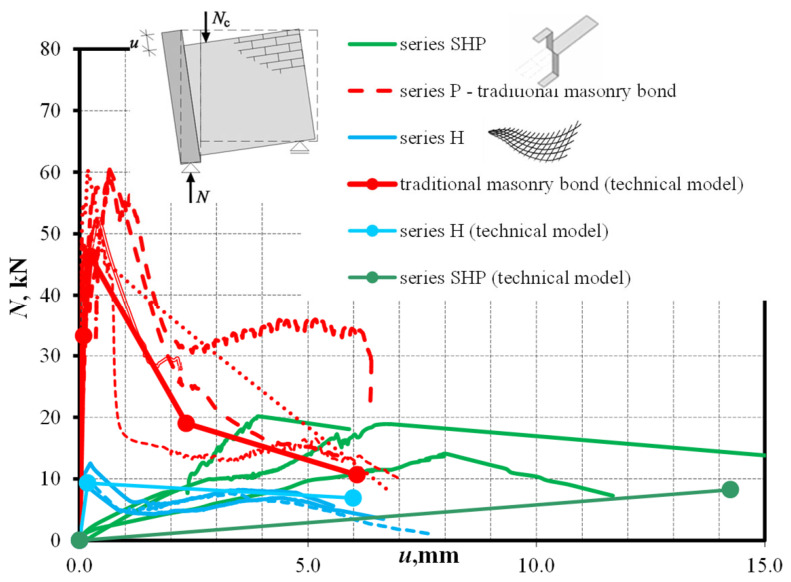
Relationship between the total force and mean relative displacement of the joint.

The behavior of the connection and its response under loading can be clearly described based on the relationship between the applied force *N* and the relative displacement *u* of the connected walls (Figure 8).

The above graph illustrates the relationship between the total force acting on the joint and the average relative displacement for three different types of connections: the traditional one (red), the connection with a rectangular mesh segment (blue), and the connection with a spatially arranged mesh (green).

The traditional connection (series P) is characterized by a high maximum force at the initial stage of loading; however, after reaching this peak, a distinct decrease in load-bearing capacity is observed. Up to the onset of cracking at the contact interface, which occurred under loads of *N_cr_* = 27.3–54.1 kN, the increase in relative displacements u remained nearly proportional to the applied load, and this stage was therefore identified as the elastic phase of joint behavior. Once cracking developed, the response entered a post-elastic regime characterized by reduced stiffness, although the joints continued to sustain load.

This stage ended at peak load levels ranging from *N_u_* = 38.6 to 59.8 kN. Further loading during the failure phase led to a noticeable decrease in forces recorded by the dynamometer, accompanied by a continued growth in relative displacements. Although the measured force values approached zero, the joint still retained a limited load-carrying capacity. In this stage, the forces were attributed to aggregate interlocking, with values of *N_ag_* = 14.1–31.1 kN.

Additional increases in joint displacements resulted in a slight rise in load and a hardening effect. The final recorded forces, referred to as residual forces, preceded ultimate failure, which involved complete separation of the connected elements and their mutual rotation. These forces ranged from *N_r_* = 8.4 to 42.9 kN.

The relationships between forces and corresponding displacements are summarized in Table 1 and Table 2. Joint stiffness in each phase was evaluated based on Equations (1)–(3), with the results presented in Table 3.

Joint stiffness in the elastic phase,

(1) $$K_{t}=\frac{N_{cr}}{u_{cr}},$$

joint stiffness in the post-elastic phase,

(2) $$K_{p}=\frac{N_{u}-N_{cr}}{u_{u}-u_{cr}},$$

joint stiffness in the failure phase,

(3) $$K_{r}=\frac{\left|N_{r}-N_{u}\right|}{u_{r}-u_{u}}.$$

The connection reinforced with mesh placed exclusively in the bed joints (series H) exhibited the lowest force values across the entire displacement range. The load–displacement curve showed considerable variability throughout the full loading range. In the initial phase, from *N* = 0 up to the load corresponding to cracking *N_cr_*, which was equal to the maximum load *N_u_*, no significant relative displacements between the connected walls were observed. This stage of behaviour was referred to as the quasi-elastic phase of the joint. The maximum force in this phase corresponded to the maximum load *N_u_* and the cracking load *N_cr_* and ranged from 9.4 to 12.5 kN. Displacements in the quasi-elastic phase were within the range 0–*u_cr_* = *u_u_*. The main factor governing the force level in the connection was friction resulting from the contact between the connected wall segments and the locally present bed-joint mortar situated in the plane of the connection.

After reaching the peak load, a rapid degradation of load capacity accompanied by increasing displacements was observed. This stage was referred to as the adjustment phase, and the forces recorded within the connection were in the range of *N_ag_* = 4.32–5.47 kN. This phase occurred within the force range *N_cr_* = *N_u_*–*N_ag_*, and the corresponding displacements were equal to *u_cr_* = *u_u_*–*u_ag_*. During this phase, the meshes embedded within the bed joints adapted to the deformed shape of the connection, while friction between the connected walls was still present.

With increasing relative displacements between the connected walls, a temporary increase in the connection force was observed. At this stage, the mesh reinforcement began to exhibit tensile tie action, and therefore this stage was defined as the tension phase.

This phase occurred within the force range *N_ag_*–*N_t_*, and the corresponding displacements were equal to *u_ag_*–*u_t_*. The peak forces resulting from the tensile action of the mesh were recorded at *N_t_* = 6.91–8.15 kN. After reaching the maximum load, a progressive degradation phase occurred, during which individual mesh fibers gradually ruptured.

The final residual forces recorded during the tests were in the range of *N_r_* = 1.04–5.55 kN. This stage was defined as the failure phase of the connection behavior. This phase occurred within the force range *N_t_*–*N_r_*, and the corresponding displacements were equal to *u_t_*–*u_r_*.

During this phase, the meshes could induce local crushing of masonry unit corners, thereby increasing the effective tensile length of the mesh reinforcement.

In the SHP series models, in which the mesh was spatially arranged, significant differences were observed compared to the H series models. During the initial quasi-elastic phase, up to the appearance of visible cracking, friction between the connected walls was lower, while the upward and downward bending of mesh fragments significantly reduced the friction effect. The forces obtained at the end of this phase ranged *N_cr_* = 8.37–9.94 kN. The forces and displacements varied within the ranges 0–*N_cr_* and 0–*u_cr_*.

An increase in the relative displacements between the connected walls resulted in an increase in the connection force. At this loading stage, similarly to the H series models, the mesh reinforcement began to exhibit tensile tie action, and this stage was therefore defined as the tension phase. The peak forces resulting from the tensile action of the mesh were recorded at *N_t_* = 12.5–15.7 kN. This phase covered the force range *N_cr_*–*N_t_* and the corresponding displacements *u_cr_*–*u_t_*.

Further increase in displacements in the SHP series specimens did not lead to degradation of the connection force, but instead caused a continued increase in load due to the tensile tie action of the meshes and friction between the connected wall segments. This phase, similarly to the elements of series H, occurred within the force range *N_t_*–*N_r_* and the corresponding displacements were equal to *u_t_*–*u_r_*.

At the end of this stage, the maximum forces *N*_tu_ ranged from 14.1 to 20.2 kN. This stage was defined as the failure phase of the connection behavior. Additionally, similarly to the H series models, the meshes could cause local crushing of masonry unit corners, thereby increasing the effective tensile length of the mesh reinforcement.

A summary of the characteristic force values, the corresponding displacements, and the calculated stiffness values of the mesh-connected models—analogously to the models without imposed constraints—is presented in Table 4, Table 5, Table 6, Table 7, Table 8 and Table 9.

The stiffness of the connections in the H and SHP series models was determined using the following relationships:

Connection stiffness in the quasi-elastic phase,

(4) $$K_{\mathrm{qe}}=\frac{N_{\mathrm{cr}}}{u_{\mathrm{cr}}},$$

Connection stiffness in the adjustment phase for the H series models,

(5) $$K_{p}=\frac{N_{cr}-N_{ag}}{u_{ag}-u_{cr}},$$

Connection stiffness in the tensile tie-action phase,

(6) $$K_{t}=\frac{N_{t}-N_{ag}}{u_{t}-u_{ag}}.$$

Connection stiffness in the failure phase,

(7) $$K_{r}=\frac{\left|N_{r}-N_{t}\right|}{u_{r}-u_{t}}.$$

From the perspective of the ultimate limit state, the maximum force values obtained in the mesh-reinforced connections were compared with those recorded for the reference models with traditional masonry bonding. In comparison with the reference models of series P, in which the connection was executed using traditional masonry bonding and the maximum force reached *N_u_* = 50.7 kN, the H series models exhibited only 22% of this load-bearing capacity, with the maximum force equal to *N_cr_* = *N_u_* = 11 kN.

Significantly better results were obtained for the SHP series models with spatially arranged meshes, in which the maximum load-bearing force reached *N_tu_* = 17.7 kN, corresponding to 35% of the force N_u_ obtained for the reference models.

Another parameter important from the serviceability point of view is the relative displacement between the connected walls. The maximum displacements obtained in the mesh-reinforced models were compared with the displacements recorded for the series P models, equal to *u_r_* =5.58 mm. In the H series models, the maximum displacements reached *u_r_* = 6.65 mm, which was approximately 19% greater than in the models with traditional masonry bonding. In the SHP series models, the maximum displacements were equal to *u_r_* = 6.25 mm, corresponding to an increase of 12% compared to the series P models.

In summary, the spatially arranged mesh connection of the SHP series exhibited significantly higher maximum forces than the connections reinforced with horizontally placed meshes of the H series. At the same time, this type of connection did not reach the load-bearing capacity corresponding to traditional masonry bonding. The utilisation of the spatial mesh configuration after cracking and after reaching the peak load is particularly important. Unlike the traditional connection, the SHP-type connection was characterised by a relatively mild stiffness degradation, which led to a more uniform stress distribution within the joint and eliminated sudden displacement jumps. As a result, the structure demonstrates a more continuous and predictable behaviour throughout the entire loading range.

It should be emphasized, however, that the traditional connection remains the reference solution and has no equivalent in terms of maximum load-bearing capacity. The models reinforced with meshes exhibited significantly lower load capacities, reduced by 65–78%, while showing only slightly greater displacements at failure, increased by 12–15%. Such alternative wall connection methods are suitable for non-structural walls, for which the required load-bearing capacity and stiffness do not need to be comparable to those of traditional solutions. The system with spatially arranged mesh constitutes an optimal solution for connections in which deformation capacity, force redistribution, and safe, progressive degradation of strength are also important. It is also worth emphasising that the proposed solution, based on spatially arranged meshes, despite its clearly lower maximum forces and higher deformability, can be used as an alternative in situations where traditional masonry bonding cannot be executed, such as when the bed joints of the connected walls do not lie in the same plane, when walls made of different materials are joined, or when geometry or construction technology prevents the use of classical bonding.

## 5. Proposal of an Analytical Model of a Connection

### 5.1. Behavior Model of the Unreinforced Connection

In study [19], a standard model describing the behavior of an unreinforced connection was proposed. The following assumptions were adopted:

(a) the nonlinear *N*–*u* relationship obtained from the experiments can be replaced by a multi-segment relationship describing all observed stages of behavior:
  i. elastic phase occurring within the load range 0–*N_cr_*,
  ii. post-elastic phase within the load range *N_cr_*–*N_u_*
  iii. failure phase within the load range *N_u_*–*N_ag_*–*N_r_*,
(b) all material parameters used in the model are determined using standard and standardized testing methods,
(c) the model is subjected to statistical validation based on the conducted experimental investigations.

Using the results of the performed model investigations [19], empirical relationships describing the connection behavior in the individual phases were summarized in Table 10.

As demonstrated in study [19], the calculated forces defining the coordinates of the individual phases of the connection behavior were, in accordance with the assumptions, lower than the forces obtained experimentally. In the case of the cracking force, the difference amounted to 15%, while for the ultimate force it was 9%. The largest discrepancies were observed in the failure phase. At this stage, the calculated values of *N_ag_* and *N_r_* were lower by 36% and 44%, respectively, compared to the mean experimental values.

In the case of relative displacements, the calculated displacement in the elastic stage differed by only 3% from the mean experimental value, whereas at the maximum force the difference exceeded 7%. In the failure stage, the displacements corresponding to the forces *N_ag_* and *N_r_* differed by 12% and 17%, respectively. The obtained results allow prediction of the forces with satisfactory accuracy and, consequently, enable proper verification of the connection under ultimate limit state (ULS) conditions. Larger discrepancies were obtained for the displacements, the knowledge of which is essential for verification under serviceability limit state (SLS) conditions. In this case, the greatest discrepancy was observed at the maximum load level.

### 5.2. Behavior Model of the Mesh-Reinforced Connection for the H Series

Following the same approach as in Section 5.1, a standard behavior model was proposed for the connection reinforced with mesh placed in the bed joints. The following assumptions were adopted:

(a) the nonlinear *N*–*u* relationship obtained from the tests can be replaced by a multi-segment relationship describing all observed phases of behavior:
  i. quasi-elastic phase occurring within the load range 0–*N_cr_* = *N_u_*,
  ii. adjustment phase, tensile tie-action phase, and failure phase occurring within the load range *N_cr_* = *N_u_*–*N_t_*–*N_r_*,
(b) all material parameters used in the model are determined using standard and standardized testing methods,
(c) an elastic–perfectly plastic model of mesh behavior is adopted,
(d) the model is statistically validated based on the conducted experimental investigations. Due to the limited number of specimens, the proposed coefficients should be regarded as preliminary calibration parameters rather than reliably validated design parameters.

**Figure 9:**
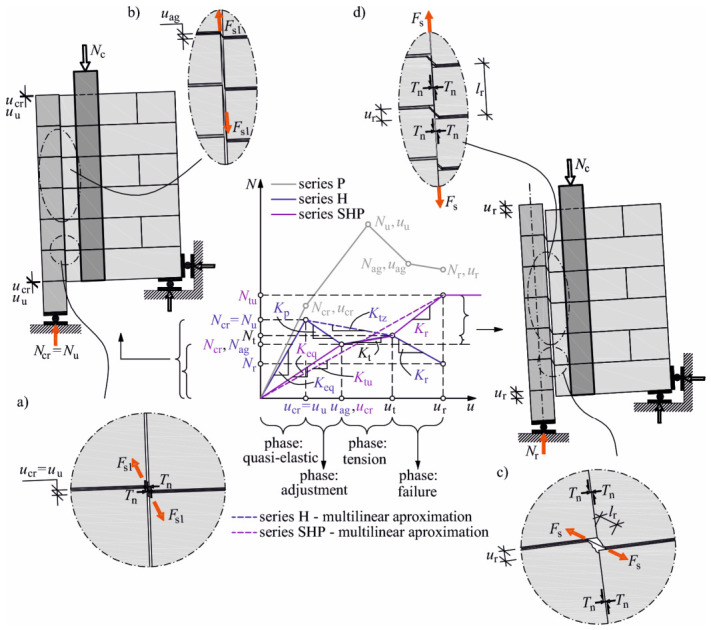
Approximation of the behavior of masonry wall connections reinforced with meshes in the H and SHP series: (**a**) horizontally arranged mesh: small forces in the mesh at the moment of cracking (quasi-elastic phase), (**b**) horizontally and spatially arranged mesh: forces in the mesh at the adjustment phase, (**c**) horizontally and spatially arranged mesh: forces in the mesh at the failure phase, (**d**) spatially arranged mesh: forces in the mesh at the failure phase.

In the quasi-elastic phase, in accordance with Figure 9a, it was assumed that, along the extension of the bed joints in which the mesh was placed, mortar is present in the interface between the connected walls and is responsible for the development of significant shear forces. In this phase, the force *F*_*s*1_ occurring in the mesh may be neglected.

Forces and displacements in the quasi-elastic phase:

(8) $$N_{cr}=N_{u}=\alpha_{eq}f_{v0}A,$$

(9) $$u_{cr}=u_{u}=\frac{N_{cr}}{K_{qe}}=\frac{N_{cr}}{\beta_{eq}K_{RL}},$$

where: *A* = 0.26 m^2^—area of the connection interface, *f_v_*_0_ = 0.31 N/mm^2^—initial shear strength of masonry, *K_RL_*= 117.1 MN/m—masonry stiffness, *α_eq_*, *β_eq_*—empirical coefficients.

In the adjustment phase, tensile tie-action phase, and failure phase (Figure 9c), within the load range *N_cr_* = *N_u_*–*N_t_*–*N_r_*_,_ the maximum force in the connection results exclusively from the tensile action of the mesh and the force *F*_s_. The forces and displacements in the combined adjustment, tensile tie-action, and failure phases are expressed by the following relationships:

(10) $$N_{t}=\alpha_{t}F_{D1}A_{M},$$

(11) $$u_{t}=\beta_{t}N_{t}l_{r}\varepsilon_{D1},$$

where: *A_M_* = 0.9 m—total width of the five mesh layers, *F*_*D*1_ = 25.1 kN/m—maximum tensile force of the mesh in the direction of the weft fibers, *l_r_* = *u_t_/ε*_*D*1_ = 0.6 *h_u_*—length of the tensioned mesh segment (Figure 9c), determined according to the relationship given in Table 6, *α_t_*, *β_t_*—empirical coefficients.

For the purpose of developing the empirical model, the limit mean values of the coefficients *α_eq_ β_eq_*, *α_t_* and *β_t_* were also determined for a significance level of *α* = 0.8 [27]. Due to the small sample size (*n* < 30), the following relationship was adopted:

(12) $$P\left(\bar{x}-t_{1-\alpha /2}\frac{S}{\sqrt{n}}<m<\bar{x}+t_{1-\alpha /2}\frac{S}{\sqrt{n}}\right)=1-\alpha ,$$

where: $\bar{x}=\sqrt{\sum \left(x-x\right)/n}$—mean value of the random sample, $S=\sqrt{\sum {\left(x-x\right)}^{2}/\left(n-1\right)}$—standard deviation of the sample,${\;t}_{1-\alpha /2}$—Student’s t-distribution statistic with n–1 degrees of freedom. The obtained lower and upper confidence interval limits for the mean coefficient values are summarized in Table 11.

As a result, using the outcomes of the performed model investigations and standard tests, the empirical relationships describing the behavior of the connection in the individual phases were summarized in Table 12, while the comparison between the calculated and experimentally obtained values is presented in Table 13.

The calculated forces defining the coordinates of the individual phases of the connection behavior were, in accordance with the assumptions, lower than the forces obtained experimentally. In the case of the cracking force corresponding to the maximum force *N_cr_* = *N_u_*, the difference amounted to 15%, while for the force *N_t_* the difference was 9%. In the case of relative displacements in the quasi-elastic stage, the calculated displacement differed by 23% from the mean experimental value, whereas at the force *N_t_* the difference exceeded 56%.

The obtained results allow for a satisfactorily accurate prediction of forces, and therefore for a correct verification of the ULS conditions for the mesh-reinforced connection with reinforcement placed in the bed joints. Larger discrepancies were observed in the case of displacements, whose estimation is essential for verifying SLS conditions. In the phases corresponding to cracking (cr) and to the maximum load (u), the predicted displacements were overestimated but remained within an acceptable range. However, at the end of the tie-action phase (t), the predicted displacement values were overly conservative; their use is safe but of limited practical value.

### 5.3. Behavior Model of the Mesh-Reinforced Connection for the SHP Series

For the connections made with spatially arranged mesh in the SHP series, a standard theoretical model of the connection behavior was also proposed, based on the following assumptions:

(a) the nonlinear N–u relationship obtained from the tests can be replaced by a single-segment relationship covering all observed phases of behavior; in the earlier phases, the mesh force Fs and friction in the contact plane are negligible, as shown in Figure 9b. These phases occur within the load range 0–*N_cr_*–*N_t_*–*N_tu_*.

The quasi-elastic phase (0–*N_cr_* and 0–*u_cr_*) omits the effects of cohesion and friction between the masonry units of the connected walls, as well as the initiation of tensile forces in the mesh. In the tension phase (*N_cr_*–*N_t_* and uc *u_cr_*–*u_t_*), changes in the inclination angle of the connected walls and friction forces are neglected. In the failure phase (*N_t_*–*N*_r_ and *u_t_*–*u_r_*), the crushing of AAC masonry units is omitted.

(b) all material parameters used in the model are determined using standard and standardized testing methods,
(c) an elastic–perfectly plastic model of mesh behavior is adopted,
(d) the model is statistically validated based on the conducted experimental investigations. Due to the limited number of specimens, the proposed coefficients should be regarded as preliminary calibration parameters rather than reliably validated design parameters.

According to Figure 9d, in the adjustment phase, tensile tie-action phase, and failure phase, within the load range *N_cr_* = *N_u_*–*N_t_*–*N_tu_*, the maximum force results exclusively from the tensile action of the mesh, represented by the force *F_s_*_1_ in Figure 9b. In this configuration, the mesh was arranged in a way that intentionally induces tensile tie action.

The forces and displacements in the combined adjustment, tensile tie-action, and failure phases are expressed by the following relationships:

(13) $$N_{tS}=\alpha_{tS}F_{D1}A_{M},$$

(14) $$u_{rS}=\beta_{tS}N_{tS}l_{r}\varepsilon_{D1},$$

where: *A_M_* = 0.36 m—total width of the 4 mesh layers, *F_D_*_1_ = 25.1 kN/m—maximum tensile force of the mesh in the direction of the weft fibers, *l_r_* = *u_t_*/*ε_D_*_1_ = *h_u_*—length of the tensioned mesh segment, *α_tS_*, *β_tS_*—empirical coefficients.

The limit mean values of the coefficients *α_tS_* and *β_tS_* for the significance level *α* = 0.8 were determined analogously to Section 5.2 using relationship (12). The obtained lower and upper confidence interval limits for the mean coefficient values are summarized in Table 14.

As a result, using the outcomes of the performed model investigations and standard tests, the empirical relationships describing the behavior of the connection in the individual phases were summarized in Table 15, while the comparison between the calculated and experimentally obtained values is presented in Table 16.

The calculated force N_tu_, defining the end of all phases of the connection behavior, was approximately 20% lower than the force obtained experimentally. In the case of relative displacements, the calculated displacements *u_r_* were 32% greater than the experimental values. The obtained results allow for a safe assessment of the connection’s performance with respect to the ultimate limit state (ULS). As in the case of the horizontally arranged meshes of the H series, larger discrepancies were observed for displacements, which are crucial for verifying SLS conditions. At the end of the tie-action phase (t), the predicted displacement values were conservative; their application is certainly safe, although it may be of limited practical use.

## 6. Conclusions

The paper presents an innovative method for connecting masonry walls. The proposed solution, based on a spatial arrangement of reinforcement mesh, constitutes an advancement over existing wall connection techniques and eliminates the identified limitations of traditional methods. In particular, it addresses the need to ensure adequate load-bearing capacity, ductility, and stability of the connection while maintaining simplicity of execution and economic efficiency.

The application of a spatial reinforcement geometry leads to a significant modification of the mechanical properties of the connection:

- in contrast to the conventional arrangement of flat mesh placed exclusively within bed joints, the proposed solution enables mechanical interlocking of the mesh elements in force transfer,
- more efficient utilization of the tensile action of the reinforcement is achieved, resulting in increased load-bearing capacity and improved deformability of the connection,
- stress concentration within the wall interface zone is reduced, which positively affects the overall durability and reliability of the system,
- the connection failure mechanism is modified: prior to failure, significant relative displacements of the connected walls within the connection zone were observed; no masonry cracking preceding failure occurred, while displacements increased rapidly with simultaneous load degradation,
- in contrast to traditional masonry connections, which are often characterized by brittle and sudden failure, the solution with spatially arranged mesh exhibits a more gradual and predictable failure process. This enables earlier identification of approaching limit states and increases structural safety through improved redistribution of internal forces,
- the proposed method constitutes an attractive and effective alternative to conventional masonry connection techniques, both in newly designed structures and in the modernization and strengthening of existing buildings. Its implementation does not require complex technologies or specialized equipment, which facilitates its practical application under construction site conditions.

Particular attention should be paid to the fact that the developed concept enables effective wall connections even when the bed joints are not located within the same plane. This feature significantly increases design flexibility and facilitates implementation under real construction conditions, where geometric and construction tolerances are unavoidable. As a result, the proposed solution may constitute a versatile engineering approach meeting contemporary requirements regarding efficiency, safety, and durability of masonry wall connections.

When using meshes placed exclusively in the bed joints and under monotonically increasing loading, the obtained force values were significantly lower than in connections with traditional masonry bonding:

- the maximum forces were lower by 78% in the H series. The mesh-reinforced models exhibited slightly greater displacements; at the maximum force level, the differences reached 19%,
- more favorable results were obtained when spatially arranged meshes were used. In the case of the maximum force, the values remained lower than those obtained for connections with traditional masonry bonding. The maximum forces were reduced by 65% in the SHP series, while the corresponding displacements were 15% greater.

The individual phases of the connection behavior were identified and defined, and on this basis an empirical approach (a connection model subjected to monotonic loading) was proposed enabling determination of the forces and displacements of wall connections using the results obtained from less complex standard tests.

Due to the limited number of test specimens in each series, differences in the conservative estimation of forces amounted to 9–15% for the H series and 20% for the SHP series. The estimated displacement values corresponding to the forces in the individual phases were greater than the experimentally determined values and ranged from 23% to 56% for the H series and approximately 32% for the SHP series. Increasing the sample size will improve the precision of estimating forces and displacements in the proposed analytical model of the joint behaviour. The current solution does not lead to unsafe results in terms of forces or displacements, particularly in the case of non-structural walls.

The most favourable agreement was obtained for the force predictions in the individual phases of behaviour, which makes the verification of the ULS conditions considerably less conservative than in the case of displacements defining the SLS condition. In this situation, the calculated displacement values significantly exceed those obtained in the experiments, and therefore the SLS assessment is markedly more conservative. The coefficients proposed in the model should thus be regarded as preliminary calibration parameters rather than reliably validated design parameters. We emphasise that the developed model has a general character, and its adaptation to other masonry technologies is possible but would require calibration of material parameters and experimental verification. In its current form, the model can be applied to AAC masonry (density class 600) constructed with system thin-layer M5 mortar and reinforced with Silica Glass Mesh.

Further research should include additional experimental models in order to statistically define the empirical parameters of the proposed models. It may then be expected that the validation process will result in significantly smaller discrepancies in the extreme values obtained.

Finite Element Method (FEM) analyses also appear necessary in order to determine the actual behavior of the connectors, particularly the effective tensile length l_r_. The target model for the SHP series connections should additionally include the earlier phases of behavior, enabling satisfactory prediction of the forces N_cr_ and N_t_ together with the corresponding displacements.

## Figures and Tables

**Table 1:** Test results for joints between unreinforced walls.

Model	Force at the Time of Cracking		Maximum Force		Aggregate Interlocking Force		Residual Force	
	*N_eri_*	*N_cr_*_,*mv*_	*N_u_*_,*i*_	*N_u_*_,*mv*_	*N_ag_*	*N_ag_*_,*mv*,*i*_	*N_r_*_,*i*_	*N_r_*_,*mv*_
	kN	kN	kN	kN	kN	kN	kN	kN
P_1	27.3	39.2	56.3	50.7	31.1	24.9	20.7	16.2
P_2	42.6		50.0		14.7		10.2	
P_3	31.2		38.6		25.5		13.8	
P_4	54.1		59.8		--		8.36	
P_5	35.1		48.1		--		--	
P_6	45.1		51.6		28.3		27.9	

**Table 2:** Test results for joints between unreinforced walls (displacements).

Model	Displacement at the Time of Cracking		Displacement Right Before Failure		Displacement at Aggregate Interlocking Force		Residual Displacement	
	*u_cr_*_,*i*_	*u_cr_*_,*mv*_	*u_u_*_,*i*_	*u_u_*_,*mv*_	*u_ag_*_,*i*_	*u_ag_*_,*mv*_	*u_r,i_*	*u_r_*_,*mv*_
	mm	mm	mm	mm	mm	mm	mm	mm
P_1	0.07	0.09	0.31	0.23	2.43	2.08	6.36	5.58
P_2	0.12		0.25		1.95		6.97	
P_3	0.12		0.16		2.22		5.64	
P_4	0.07		0.17		--		6.72	
P_5	0.06		0.10		--		--	
P_6	0.08		0.36		1.71		2.22	

**Table 3:** Test results for joints between unreinforced walls (joint stiffness).

Model	Elastic Joint Stiffness		Post-Elastic Joint Stiffness		Residual Joint Stiffness	
	*K_t_*_,*i*_	*K_t_*_,*mv*_	*K_p_*_,*i*_	*K_p_*_,*mv*_	*K_r_*_,*i*_	*K_r_*_,*mv*_
	MN/m	MN/m	MN/m	MN/m	MN/m	MN/m
P_1	413	496	119	123	5.89	7.39
P_2	341		60		5.93	
P_3	268		163		4.51	
P_4	804		52.8		7.86	
P_5	562		322		--	
P_6	590		23		12.75	

**Table 4:** Test results for joints between mesh-connected H series.

Model	Force at the Time of Cracking and Maximum Force		Aggregate Interlocking Force		Tensile Tie Force		Residual Force	
	*N_cr_*_,*i*_ *N_u_*_,*i*_	*N_cr_*_,*mv*_ *N_u_*_,*mv*_	*N_ag_*_,*i*_	*N_ag_*_,*mv*_	*N_t_*_,*i*_	*N_t_*_,*mv*_	*N_r_*_,*i*_	*N_r_*_,*mv*_
	kN	kN	kN	kN	kN	kN	kN	kN
H_1	9.43	11.0	4.32	4.80	6.91	7.56	3.49	3.36
H_2	11.0		4.61		7.63		1.04	
H_3	12.5		5.47		8.15		5.55	

**Table 5:** Test results for joints between mesh-connected SHP series.

Model	Force at the Time of Cracking		Tensile Tie Force		Maximum Force	
	*N_cr_*_,*i*_	*N_cr_*_,*mv*_	*N_t_*_,*i*_	*N_t_*_,*mv*_	*N_tu_*_,*i*_	*N_tu_*_,*mv*_
	kN	kN	kN	kN	kN	kN
SHP_1	9.72	9.34	12.8	13.6	14.1	17.7
SHP_2	8.37		12.5		20.2	
SHP_3	9.94		15.7		18.9	

**Table 6:** Test results for joints between mesh-connected walls (displacements) H series.

Model	Displacement at the Time of Cracking and Displacement Right Before Failure		Displacement at Aggregate Interlocking Force		Displacements Corresponding to the Tensile Tie Force		Residual Displacement		Length of the Tensioned Mesh Segment	
	*u_cr_*_,*i*_ *u_u_*_,*i*_	*u_cr_*_,*mv*_ *u_u_*_,*mv*_	*u_ag_*_,*i*_	*u_ag_*_,*mv*_	*u_t,i_*	*u_t_*_,*mv*_	*u_r_*_,*i*_	*u_r_*_,*mv*_	*l_r,i_*	*l_r_*_,*mv*_
	mm	mm	mm	mm	mm	mm	mm	mm	mm	mm
H_1	0.0966	0.143	1.60	1.42	4.41	3.85	6.64	6.65	222	143 (~0.6h_u_)
H_2	0.0939		1.15		3.46		7.70		135	
H_3	0.238		1.52		3.69		5.62		73.7	

**Table 7:** Test results for joints between mesh-connected walls (displacements) SHP series.

Model	Displacement at the Time of Cracking		Displacements Corresponding to the Tensile Tie Force		Residual Displacement	
	*u_cr_*_,*i*_	*u_cr_*_,*mv*_	*u_t_*_,*i*_	*u_t_*_,*mv*_	*u_r_*_,*i*_	*u_r_*_,*mv*_
	mm	mm	mm	mm	mm	mm
SHP_1	5.01	3.40	7.44	5.37	8.01	6.25
SHP_2	2.23		2.81		3.91	
SHP_3	2.95		5.87		6.84	

**Table 8:** Test results for joints between mesh-connected walls. (joint stiffness) H series.

Model	Quasi-Elastic Stiffness		Stiffness in the Adjustment Phase		Stiffness in the Tensile Tie-Action Phase		Stiffness in the Failure Phase	
	*K_qe_*_,*i*_	*K_qe_*_,*mv*_	*K_p_*_,*i*_	*K_p_*_,*mv*_	*K_t_*_,*i*_	*K_t_*_,*mv*_	*K_r_*_,*i*_	*K_r_*_,*mv*_
	MN/m	MN/m	MN/m	MN/m	MN/m	MN/m	MN/m	MN/m
H_1	97.6	89.2	3.40	4.99	0.924	1.15	1.53	1.48
H_2	117		6.07		1.31		1.55	
H_3	52.6		5.49		1.24		1.34	

**Table 9:** Test results for joints between mesh-connected walls. (joint stiffness) SHP series.

Model	Quasi-Elastic Stiffness		Stiffness in the Tensile Tie-Action Phase		Stiffness in the Failure Phase	
	*K_qe,i_*	*K_qe,mv_*	*K_t,i_*	*K_t,mv_*	*K_r,i_*	*K_r,mv_*
	MN/m	MN/m	MN/m	MN/m	MN/m	MN/m
SHP_1	1.94	3.02	1.26	3.43	2.31	4.23
SHP_2	3.75		7.07		7.03	
SHP_3	3.37		1.96		3.34	

**Table 10:** Summary of relationships describing the behavior of the unreinforced wall connection.

Connection Behavior Phase	Force	Stiffness	Displacement
Elastic phase	$N_{cr}=0.67\tau_{u,RL}A$	$K_{t}=3.22K_{RL}$	$u_{cr}=N_{cr}/3.22K_{RL}$
Post-elastic phase	$N_{u}=0.91\tau_{u,RL}A$	$K_{p}=0.14K_{RL}$	$u_{u}=\left(N_{u}-N_{cr}\right)/0.14K_{RL}$
Failure phase	$N_{ag}=0.37\tau_{u,RL}A$	$K_{ag}=\left(N_{u}-N_{ag}\right)/\left(u_{u}-u_{ag}\right)$	$u_{ag}=5.39\tau_{u,RL}A/K_{RL}$
	$N_{r}=0.21\tau_{u,RL}A$	$K_{r}=\left(N_{u}-N_{r}\right)/\left(u_{r}-u_{u}\right)$	$u_{r}=\frac{2G_{f}^{II}A-N_{u}\left(u_{ag}-u_{u}\right)+N_{ag}u_{u}+N_{r}\left(u_{ag}-2u_{u}\right)}{\left(N_{ag}-N_{r}\right)}$

where: τ_*cr,RL*_ = 0.192 MPa, τ_*u,RL*_ = 0.196 N/mm^2^, *K*_*RL*_ = 117.7 MN/m.

**Table 11:** Validation of the empirical coefficients of the wall connection model for the H series mesh-reinforced connections.

Model	*x*_i_			
	$\alpha_{eq,i}=\frac{N_{cr,i}}{f_{v0}A}$	$\beta_{eq,i}=\frac{K_{qe,i}}{K_{RL}}$	$\alpha_{t,i}=\frac{N_{t,i}}{F_{D1}A_{M}}$	$\beta_{t,i}=\frac{u_{t,i}}{\alpha_{t,i}F_{D1}A_{M}l_{r,i}\varepsilon_{D1}}$
H_1	0.12	0.834	0.306	0.145
H_2	0.14	1.00	0.338	0.169
H_3	0.15	0.45	0.361	0.308
n	3	3	3	3
$\bar{x}$	0.14	0.762	0.335	0.207
S	0.019	0.283	0.027	0.088
$t_{1-\alpha /2}$	1.89	1.89	1.89	1.89
$\bar{x}-t_{1-\alpha /2}\frac{S}{\sqrt{n}}$	0.115	0.454	0.305	0.111
$\bar{x}+t_{1-\alpha /2}\frac{S}{\sqrt{n}}$	0.16	1.07	0.365	0.304

**Table 12:** Summary of relationships describing the behavior of wall connections reinforced with meshes in the H series.

Connection Behavior Phase	Force	Stiffness	Displacement
Quasi-elastic phase	$N_{cr}=N_{u}=0.115f_{v0}A$	$K_{eq}=0.454K_{RL}$	$u_{cr}=u_{u}=\frac{N_{cr}}{0.454K_{RL}}$
Adjustment, tensile tie-action, and failure phases	$N_{t}=0.305F_{D1}A_{M}$	$K_{t}=\frac{15}{h_{u}\varepsilon_{D1}}$	$u_{t}=0.304N_{t}l_{r}\varepsilon_{D1}$

**Table 13:** Comparison of experimental and calculated results according to the standard model for wall connections reinforced with meshes in the H series.

Experimental Results		Calculated Results	
Forces		Forces	
***N_cr,mv_*** ***N_u,mv_*** **kN**	***N_t,mv_*** **kN**	***N_cr,cal_*** **kN**	***N_t,cal_*** **kN**
11.0	7.55	9.34	6.89
Displacements		Displacements	
*u_cr,mv_* *u_u,mv_* mm	*u_t,mv_* mm	*u_cr,cal_* mm	*u_t,cal_* mm
0.143	3.85	0.176	5.99

**Table 14:** Validation of the empirical coefficients of the wall connection model for the SHP series mesh-reinforced connections.

Model	*x_i_*	
	$\alpha_{tS,i}=\frac{N_{tu,i}}{F_{D1}A_{M}}$	$\beta_{tS,i}=\frac{u_{r,i}}{\alpha_{tS,i}F_{D1}A_{M}l_{r,i}\varepsilon_{D1}}$
SHP_1	1.56	0.119
SHP_2	2.23	0.04056
SHP_3	2.09	0.0758
n	3	3
$\bar{x}$	1.96	0.0784
S	0.354	0.0392
$t_{1-\alpha /2}$	1.89	1.89
$\bar{x}-t_{1-\alpha /2}\frac{S}{\sqrt{n}}$	1.58	0.0357
$\bar{x}+t_{1-\alpha /2}\frac{S}{\sqrt{n}}$	2.35	0.121

**Table 15:** Summary of relationships describing the behavior of wall connections reinforced with meshes in the SHP series.

Connection Behavior Phase	Force	Stiffness	Displacement
Adjustment, tensile tie-action, and failure phases	$N_{tu}=1.58F_{D1}A_{M}$	$K_{tu}=\frac{8.3}{h_{u}\varepsilon_{D1}}$	$u_{r}=0.121N_{tu}l_{r}\varepsilon_{D1}$

**Table 16:** Comparison of experimental and calculated results according to the standard model for wall connections reinforced with meshes in the SHP series.

Experimental Results	Calculated Results
Forces	Forces
***N_tu,mv_*** **kN**	***N_tu,cal_*** **kN**
17.7	14.3
Displacements	Displacements
*u_r,mv_* mm	*u_r,cal_* mm
6.25	8.24

## Data Availability

The original contributions presented in this study are included in the article material. Further inquiries can be directed to the corresponding author.
